# Effects of Curcumin Nanoparticles in Isoproterenol-Induced Myocardial Infarction

**DOI:** 10.1155/2019/7847142

**Published:** 2019-05-07

**Authors:** Paul-Mihai Boarescu, Ioana Chirilă, Adriana E. Bulboacă, Ioana Corina Bocșan, Raluca Maria Pop, Dan Gheban, Sorana D. Bolboacă

**Affiliations:** ^1^Department of Pathophysiology, Iuliu Haţieganu University of Medicine and Pharmacy Cluj-Napoca, 400012 Cluj-Napoca, Romania; ^2^Department of Medical Informatics and Biostatistics, Iuliu Haţieganu University of Medicine and Pharmacy Cluj-Napoca, 400349 Cluj-Napoca, Romania; ^3^County Clinical Emergency Hospital of Cluj-Napoca, 400006 Cluj-Napoca, Romania; ^4^Department of Pharmacology, Toxicology and Clinical Pharmacology, Iuliu Haţieganu University of Medicine and Pharmacy Cluj-Napoca, 400337 Cluj-Napoca, Romania; ^5^Department of Pathological Anatomy, Iuliu Haţieganu University of Medicine and Pharmacy Cluj-Napoca, 400006 Cluj-Napoca, Romania

## Abstract

Curcumin has anti-inflammatory, antioxidative, anticarcinogenic, and cardiovascular protective effects. Our study is aimed at evaluating the effects of pretreatment with curcumin nanoparticles (CCNP) compared to conventional curcumin (CC) on isoproterenol (ISO) induced myocardial infarction (MI) in rats. Fifty-six Wistar-Bratislava white rats were randomly divided into eight groups of seven rats each. Curcumin and curcumin nanoparticles were given by gavage in three different doses (100 mg/kg body weight (bw), 150 mg/kg bw, and 200 mg/kg bw) for 15 days. The MI was induced on day 13 using 100 mg/kg bw ISO administered twice, with the second dose 24 h after the initial dose. The blood samples were taken 24 h after the last dose of ISO. The antioxidant, anti-inflammatory, and cardioprotective effects were evaluated in all groups. All doses of CC and CCNP offered a cardioprotective effect by preventing creatine kinase-MB leakage from cardiomyocytes, with the best result for CCNP. All the oxidative stress parameters were significantly improved after CCNP compared to CC pretreatment. CCNP was more efficient than CC in limiting the increase in inflammatory cytokine levels (such as TNF-*α*, IL-6, IL-1*α*, IL-1*β*, MCP-1, and RANTES) after MI. MMP-2 and MMP-9 levels decreased more after pretreatment with CCNP than with CC. CCNP better prevented myocardial necrosis and reduced interstitial edema and neutrophil infiltration than CC, on histopathological examination. Therefore, improving the bioactivity of curcumin by nanotechnology may help limit cardiac injury after myocardial infarction.

## 1. Introduction

Over the last decade, cardiovascular diseases have become the most important cause of death worldwide and in many high-income countries during the past century; now, low- and middle-income countries are seeing an alarming and accelerating increase in cardiovascular disease rates [1]. Coronary heart diseases often occur at a lower prevalence rate than stroke and account for 10% to 35% of deaths, but still, in 2010, they caused an estimated 16 million deaths and led to 293 million disability-adjusted life years lost [1]. Efforts to improve the acute management of myocardial infarction (MI) led to the application of lifesaving interventions such as drug therapies, percutaneous coronary interventions, and strategies to both primary and secondary preventions by reducing deaths caused by cardiovascular diseases [1]. Acute myocardial infarction is defined as the necrosis of cardiomyocytes due to prolonged myocardial ischemia and leads to an imbalance between coronary blood supply and myocardial demand [2]. The acute myocardial infarction is associated with an inflammatory response, an alteration of the extracellular matrix due to the release of free radicals and proteolytic enzymes, which progresses towards remodeled myocardium [2]. The inflammatory process can influence the extent of the myocardial lesions, as previously showed [3, 4]. The use of anti-inflammatory drugs in myocardial ischemia may reduce the extent of ischemic lesions [4]. Furthermore, the treatment with antioxidants can exert cardioprotective effects by reducing the oxidative stress during myocardial ischemia and reperfusion injury [5]. Isoproterenol (ISO), a *β*-adrenoceptor agonist, can, in high doses, induce myocardial infarction (MI) [6]. ISO generates through autooxidation highly cytotoxic free radicals that stimulate the peroxidation of membrane phospholipids leading to severe damage to the myocardial membrane [7]. Curcumin has been previously used to treat a variety of diseases in Asian traditional medicine, including colon or pancreatic cancer, rheumatoid arthritis, vitiligo, psoriasis, diabetes mellitus, and cognitive dysfunctions [8, 9]. Goel and coauthors demonstrated the anti-inflammatory, antioxidative, anticarcinogenic, and cardiovascular protective effects of curcumin [8, 10]. Curcumin also improves systolic dysfunction and prevents cardiac remodeling after myocardial infarction [9, 11, 12]. The molecular targets of curcumin are growth factors, transcription factors, and their receptors, genes, enzymes, cytokines, and cells regulating proliferation and apoptosis [9, 11]. The cardioprotective effect of curcumin has been associated with the attenuation of the oxidative stress and the activity of the active matrix metalloproteinases [11]. Curcumin also inhibits the differentiation of cardiac fibroblasts and maintains the balance between collagen degradation and synthesis [9, 11]. After oral administration, curcumin has a very poor absorption due to its hydrophobic characteristics, and the reduced oral bioavailability may impede its proper use [13, 14].

Our study investigated the effects of pretreatment with curcumin nanoparticles compared to conventional curcumin on the changes in oxidative parameters, inflammatory cytokine, and matrix metalloproteinase levels during ISO-induced MI in rats.

## 2. Material and Methods

### 2.1. Ethics Statement

The experimental protocol followed the Helsinki Declaration on animal studies and was approved by the Ethics Committee of the Iuliu Hațieganu University of Medicine and Pharmacy Cluj-Napoca (53/22.01.2018) and by the Sanitary-Veterinary and Food Safety Directorate from Cluj-Napoca (99/21.02.2018). All national and international guidelines for the care and use of animals were closely followed.

### 2.2. Drugs and Chemicals

Isoproterenol hydrochloride (ISO) and curcumin (CC) (≥94% curcuminoid content and ≥80% curcumin) were purchased from Sigma-Aldrich (St. Louis, USA). Curcumin nanoparticles (CCNP) were obtained from CVI Pharma (Vietnam). In the CCNP, the active ingredient, curcumin, is enclosed in polymer-based nanoparticles of size from 30 nm to 100 nm. Curcumin nanoparticles were prepared with high-frequency ultrasonic waves to transform curcumin into nanosized molecules. Biocompatible water-based polymers were used to protect curcumin particles well dispersed in water and to assure an increase in absorption (up to 95%). All other chemicals used were of analytical grade.

### 2.3. Experimental Model

Fifty-six Wistar-Bratislava white female rats, weighing between 200 and 250 grams, from the Animal Department of Faculty of Medicine, Iuliu Haţieganu University of Medicine and Pharmacy Cluj-Napoca, were kept in polypropylene cages, acclimated at standard environmental conditions of 25 ± 2°C, 50 ± 15% humidity, and a natural light-dark cycle at the Department of Pathophysiology. Animals had free access to standard pellets (Cantacuzino Institute, Bucharest, Romania) and water *ad libitum*.

The rats were randomly divided into eight groups of seven rats/group as presented in Table 1.

The dose of 100 mg/kg bw of ISO was previously demonstrated to cause ECG, biological, and histopathological changes, characteristics for MI [6].

The curcumin and curcumin nanoparticle doses have been chosen for their myocardial protection potential in acute infarction, based on previously reported results [15–17].

In our study, CC and CCNP dissolved in peanut oil were administered by gavage for 15 days. On days 13 and 14, groups two to eight (Table 1) received ISO (100 mg/kg bw s.c.) once daily (with the second dose 24 hours after the initial dose), for the induction of myocardial infarction following the model described by Tanwar and coauthors [16]. The rats in the control group (group 1, Table 1) were injected saline subcutaneously following the schedule of the pretreated groups.

### 2.4. Blood Samples and Serum Analysis

On day 15, 24 hours after the last dose of ISO, the rats were placed under general anesthesia with ketamine and xylazine; blood samples were collected from the retroorbital plexus; afterward, the rats were sacrificed by an overdose of anesthetics. The serum levels of two enzymes (namely, creatine kinase (CK) and creatine kinase-MB (CK-MB)) and five oxidative stress parameters (namely, malondialdehyde (MDA), thiol, the indirect assessment of NO synthesis (NOx), total oxidative status (TOS), and total antioxidative capacity (TAC)) were measured using the Jasco V-530 UV-Vis spectrophotometer (Jasco International Co. Ltd., Tokyo, Japan). The serum levels of six inflammatory cytokines (namely, tumor necrosis factor alpha (TNF-*α*), interleukin- (IL-) 1*α*, IL-1*β*, IL-6, monocyte chemoattractant protein-1 (MCP1), and regulated upon activation, normal T cell expressed and secreted (RANTES)) (Signosis Inc., Santa Clara, CA, USA) and of two matrix metalloproteinases (namely, 2 and 9 (MMP-2 and MMP-9)) (Boster Biological Technology Co. Ltd., California, USA) were also measured using the ELISA technique (Stat Fax 303 Plus Microstrip Reader, Minneapolis, USA).

### 2.5. Histopathological Examination

The hearts of the rats included in the study were excised, washed immediately with saline, and then fixed in 10% formalin. Tissues were embedded in paraffin, sectioned at 3 *μ*m, and stained with hematoxylin and eosin (H&E). The sections were examined under a light microscope, and then photomicrographs at ×400 magnification were taken.

### 2.6. Statistical Analysis

Statistical analyses were done with Statistica 8 (v. 8, StatSoft, USA). The measured data were expressed as mean and standard deviation. The differences between groups in oxidative stress parameters, cytokines, and metalloproteinases levels were assessed with the Mann-Whitney test. The distribution of investigated markers in groups was plotted as individual values (circles) and the median (line) as recommended by Weissgerber and coauthors [18]. The level of significance was set at a *p* value < 0.05.

## 3. Results

No rats were lost from the follow-up, and the analysis was performed on all seven rats in each group. MI was successfully induced after ISO administration, demonstrated by the elevation of CK and CK-MB. All *p* values are presented in Supplementary Table 1.

### 3.1. Evaluation of Serum Levels of Myocardial Infarction Enzymes

**Figure 1:**
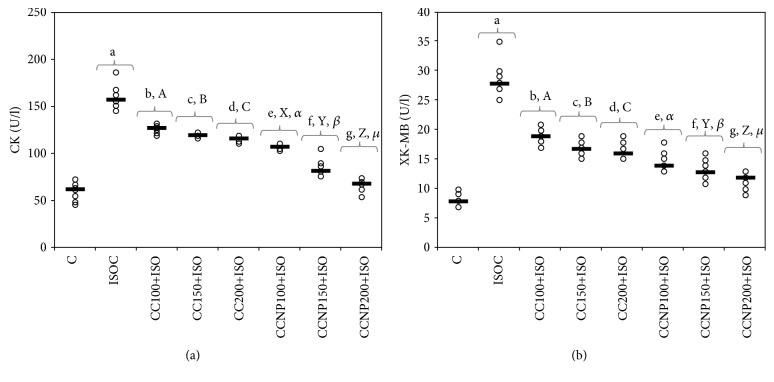
Distribution of serum levels of myocardial infarction enzymes ((a) CK (creatine kinase) and (b) CK-MB (creatine kinase-MB)) by groups. The horizontal line is given by the median, and the circles represent the individual values. C = control; ISOC = isoproterenol without any pretreatment; CC = curcumin solution, in doses of 100 mg/kg bw (CC100), 150 mg/kg bw (CC150), and 200 mg/kg bw (CC200); CCNP = curcumin nanoparticle solution, in doses of 100 mg/kg bw (CCNP100), 150 mg/kg bw (CCNP150), and 200 mg/kg bw (CCNP200). The Roman and Greek letters correspond to the *p* values < 0.03: ^a^ISOC compared to C, ^b^CC100+ISO compared to ISOC, ^c^CC150+ISO compared to ISOC, ^d^CC200+ISO compared to ISOC, ^e^CCNP100+ISO compared to ISOC, ^f^CCNP150+ISO compared to ISOC, ^g^CCNP200+ISO compared to ISOC, ^A^CC100+ISO compared to CC150+ISO, ^B^CC150+ISO compared to CC200+ISO, ^C^CC100+ISO compared to CC200+ISO, ^X^CCNP100+ISO compared to CCNP150+ISO, ^Y^CCNP150+ISO compared to CCNP200+ISO, ^Z^CCNP100+ISO compared to CCNP200+ISO, *^α^*CC100+ISO compared to CCNP100+ISO, *^β^*CC150+ISO compared to CCNP150+ISO, and *^μ^*CC200+ISO compared to CCNP200+ISO.

Administration of ISO led to increased serum levels of CK and CK-MB (Table 2 and Figure 1). The increase in dose better prevented the elevation of CK not only for CC but also for CCNP, with best results for CCNP (Table 2 and Figure 1(a), *p* < 0.03). Best effect in reducing CK-MB levels after MI induction for CC was obtained for the dose of 200 mg/kg bw. Similar results were obtained for the doses of 100 and 150 mg/kg bw CCNP on CK-MB levels (Table 2 and Figure 1(b), *p* > 0.05). Pretreatment with CCNP in all doses had a better effect compared to that with CC in the same doses (Table 2 and Figure 1(b), *p* < 0.03).

### 3.2. Assessment of Oxidative Stress Parameters

**Figure 2:**
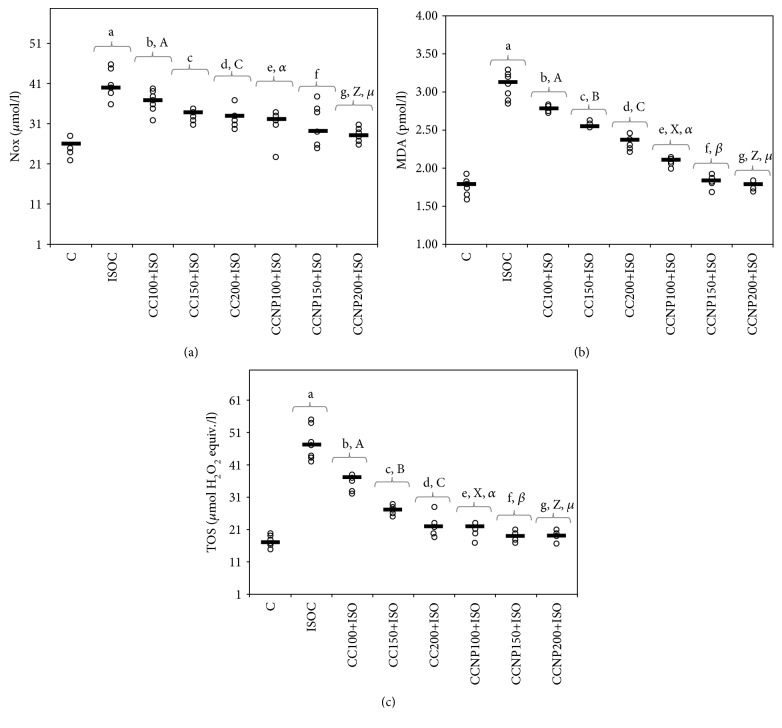
Distribution of oxidative stress intensity ((a) NOx (nitric oxide), (b) MDA (malondialdehyde), and (c) TOS (total oxidative status)) by groups. The horizontal line is given by the median, and the circles represent the individual values. C = control; ISOC = isoproterenol without any pretreatment; CC = curcumin solution, in doses of 100 mg/kg bw (CC100), 150 mg/kg bw (CC150), and 200 mg/kg bw (CC200); CCNP = curcumin nanoparticle solution, in doses of 100 mg/kg bw (CCNP100), 150 mg/kg bw (CCNP150), and 200 mg/kg bw (CCNP200). The Roman and Greek letters correspond to the *p* values < 0.05: ^a^ISOC compared to C, ^b^CC100+ISO compared to ISOC, ^c^CC150+ISO compared to ISOC, ^d^CC200+ISO compared to ISOC, ^e^CCNP100+ISO compared to ISOC, ^f^CCNP150+ISO compared to ISOC, ^g^CCNP200+ISO compared to ISOC, ^A^CC100+ISO compared to CC150+ISO, ^B^CC150+ISO compared to CC200+ISO, ^C^CC100+ISO compared to CC200+ISO, ^X^CCNP100+ISO compared to CCNP150+ISO, ^Z^CCNP100+ISO compared to CCNP200+ISO, *^α^*CC100+ISO compared to CCNP100+ISO, *^β^*CC150+ISO compared to CCNP150+ISO, and *^μ^*CC200+ISO compared to CCNP200+ISO.

The induction of MI resulted in an elevation in oxidative stress markers (Table 3). Higher doses of CC proved more efficient in preventing the increase in MDA and TOS (*p* ≤ 0.0152). Compared to the CC, CCNP prevented the elevation in MDA (*p* ≤ 0.0017, Table 3 and Figure 2(b)), TOS (*p* ≤ 0.0298, Table 3 and Figure 2(c)), and NOx at doses of 100 mg/kg bw (*p* = 0.0088) and 200 mg/kg bw (*p* = 0.004). No differences were found between the CCNP doses of 100 mg/kg bw and 150 mg/kg bw in preventing the NOx elevation (*p* > 0.9999, Table 3 and Figure 2(a)). Both 150 and 200 mg/kg bw CCNP doses had a similar effect on MDA, TOS, and NOx levels (*p* > 0.05, Table 3 and Figures 2(a)–2(c)).

**Figure 3:**
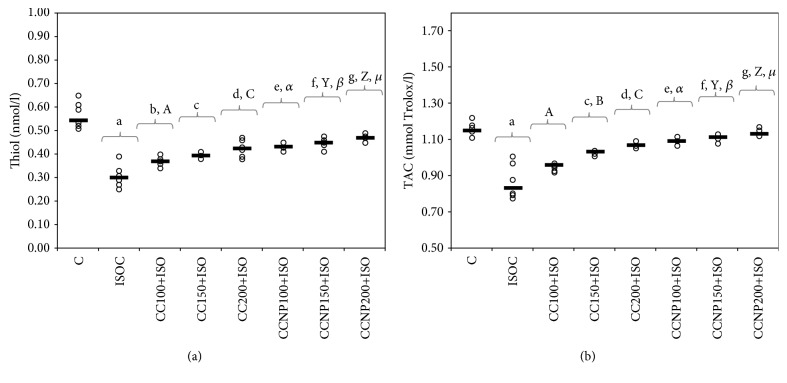
Distribution of antioxidant capacity ((a) thiol and (b) TAC (total antioxidant capacity)) by groups. C = control; ISOC = isoproterenol without any pretreatment; CC = curcumin solution, in doses of 100 mg/kg bw (CC100), 150 mg/kg bw (CC150), and 200 mg/kg bw (CC200); CCNP = curcumin nanoparticle solution, in doses of 100 mg/kg bw (CCNP100), 150 mg/kg bw (CCNP150), and 200 mg/kg bw (CCNP200). The Roman and Greek letters correspond to the *p* values < 0.05: ^a^ISOC compared to C, ^b^CC100+ISO compared to ISOC, ^c^CC150+ISO compared to ISOC, ^d^CC200+ISO compared to ISOC, ^e^CCNP100+ISO compared to ISOC, ^f^CCNP150+ISO compared to ISOC, ^g^CCNP200+ISO compared to ISOC, ^A^CC100+ISO compared to CC150+ISO, ^B^CC150+ISO compared to CC200+ISO, ^C^CC100+ISO compared to CC200+ISO, ^Y^CCNP150+ISO compared to CCNP200+ISO, ^Z^CCNP100+ISO compared to CCNP200+ISO, *^α^*CC100+ISO compared to CCNP100+ISO, *^β^*CC150+ISO compared to CCNP150+ISO, and *^μ^*CC200+ISO compared to CCNP200+ISO.

The induction of myocardial infarction was associated with a significant decrease in both thiol and TAC values (Table 4 and Figures 3(a) and 3(b)). Pretreatment with any CC dose prevented the reduction in thiol levels. Serum thiol levels were higher after CCNP than after CC pretreatment (*p* ≤ 0.0152, Table 4 and Figure 3(a)). TAC significantly increased after the use of the highest CC and all CCNP doses, but with higher levels in groups treated with CCNP (*p* ≤ 0.0152, Table 4 and Figure 3(b)). A similar effect of preventing the reduction in antioxidant capacity was observed for CCNP at doses of 100 and 150 mg/kg bw (*p* > 0.05, Table 4 and Figure 3(b)).

### 3.3. Evaluation of Serum Cytokine Levels

**Figure 4:**
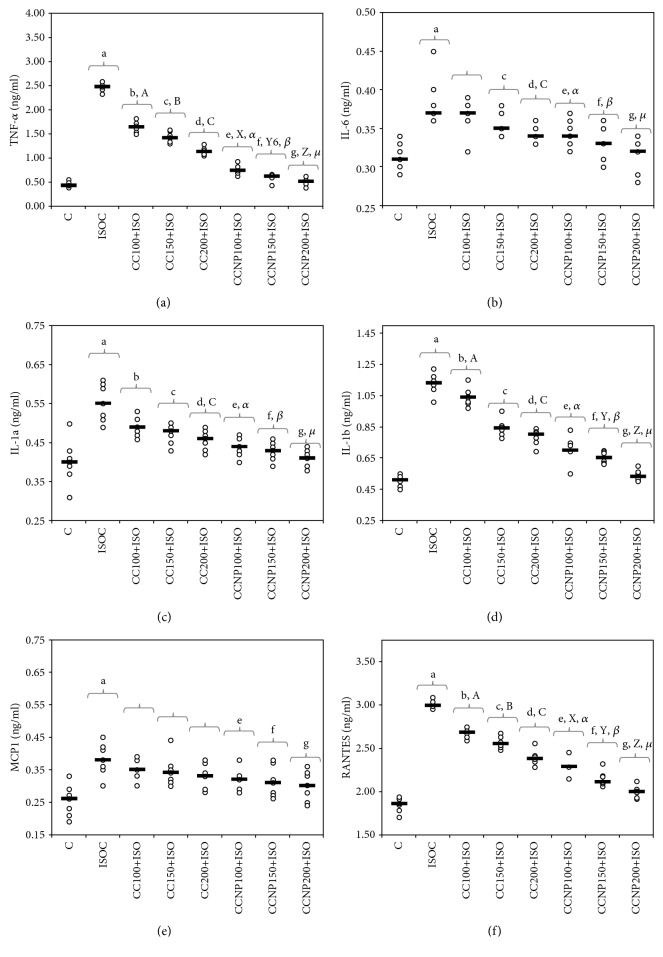
Distribution of serum cytokine levels ((a) TNF-*α* (tumor necrosis factor alpha), (b) IL-6 (interleukin 6), (c) IL-1*α* (interleukin 1a), (d) IL-1*β* (interleukin 1*β*), (e) MCP1 (monocyte chemoattractant protein-1), and (f) RANTES (regulated upon activation, normal T cell expressed and secreted)) by groups. C = control; ISOC = isoproterenol without any pretreatment; CC = curcumin solution, in doses of 100 mg/kg bw (CC100), 150 mg/kg bw (CC150), and 200 mg/kg bw (CC200); CCNP = curcumin nanoparticle solution, in doses of 100 mg/kg bw (CCNP100), 150 mg/kg bw (CCNP150), and 200 mg/kg bw (CCNP200). The Roman and Greek letters correspond to the *p* values < 0.05: ^a^ISOC compared to C, ^b^CC100+ISO compared to ISOC, ^c^CC150+ISO compared to ISOC, ^d^CC200+ISO compared to ISOC, ^e^CCNP100+ISO compared to ISOC, ^f^CCNP150+ISO compared to ISOC, ^g^CCNP200+ISO compared to ISOC, ^A^CC100+ISO compared to CC150+ISO, ^B^CC150+ISO compared to CC200+ISO, ^C^CC100+ISO compared to CC200+ISO, ^X^CCNP100+ISO compared to CCNP150+ISO, ^Y^CCNP150+ISO compared to CCNP200+ISO, ^Z^CCNP100+ISO compared to CCNP200+ISO, *^α^*CC100+ISO compared to CCNP100+ISO, *^β^*CC150+ISO compared to CCNP150+ISO, and *^μ^*CC200+ISO compared to CCNP200+ISO.

The serum levels of TNF-*α*, IL-6, IL-1*α*, IL-1*β*, MCP-1, and RANTES increased after the induction of myocardial infarction (Table 5 and Figures 4(a)–4(f)). All CC and CCNP doses used prevented the increase in TNF-*α*, IL-1*α*, IL-1*β*, and RANTES, but better results were observed for CCNP as compared to CC (*p* ≤ 0.0409, Table 5 and Figures 4(a), 4(c), 4(d), and 4(f)). Curcumin at a dose of 100 mg/kg bw did not prevent the increase in IL-6 (*p* = 0.7983, Table 5 and Figure 4(b)). All doses of CCNP had a similar effect regarding the prevention of IL-6 elevation (*p* > 0.05, Table 5 and Figure 4(b)) with a significantly better effect than CC (*p* ≤ 0.0409). CC in doses of 150 mg/kg bw and 100 mg/kg bw had a similar effect on IL-6 and IL-1*α* (*p* > 0.05, Table 5 and Figures 4(b) and 4(c)), while a dose of 200 mg/kg bw CC provided no added benefit over the 150 mg/kg bw dose CC for IL-6, IL-1*α*, and IL-1*β*. CCNP in doses of 100 and 150 mg/kg bw had a similar effect on the levels of IL-1*α*, and IL-1*β* (*p* > 0.05, Table 5 and Figures 4(c) and 4(d)), while CCNP in 200 mg/kg bw doses provided similar results on IL-1*α* levels to 100 mg/kg bw CCNP (*p* > 0.05, Table 5 and Figure 4(c)). The levels of MCP-1 after MI were reduced by CCNP at the highest doses (with no significant differences between doses *p* > 0.05) but were not influenced by CC (Table 5 and Figure 4(e)).

### 3.4. Evaluation of Serum Matrix Metalloproteinases

**Figure 5:**
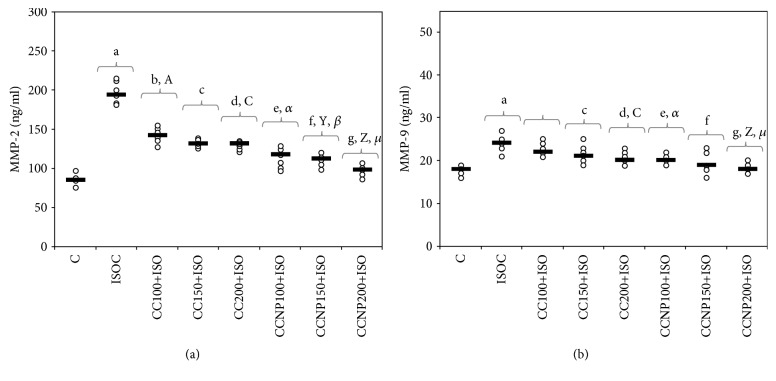
Distribution of serum matrix metalloproteinases ((a) MMP-2 (matrix metalloproteinase-2) and (b) MMP-9 (matrix metalloproteinase-9)) per group. C = control; ISOC = isoproterenol without any pretreatment; CC = curcumin solution, in doses of 100 mg/kg bw (CC100), 150 mg/kg bw (CC150), and 200 mg/kg bw (CC200); CCNP = curcumin nanoparticle solution, in doses of 100 mg/kg bw (CCNP100), 150 mg/kg bw (CCNP150), and 200 mg/kg bw (CCNP200). The Roman and Greek letters correspond to the *p* values < 0.05: ^a^ISOC compared to C, ^b^CC100+ISO compared to ISOC, ^c^CC150+ISO compared to ISOC, ^d^CC200+ISO compared to ISOC, ^e^CCNP100+ISO compared to ISOC, ^f^CCNP150+ISO compared to ISOC, ^g^CCNP200+ISO compared to ISOC, ^A^CC100+ISO compared to CC150+ISO, ^C^CC100+ISO compared to CC200+ISO, ^X^CCNP100+ISO compared to CCNP150+ISO, ^Z^CCNP100+ISO compared to CCNP200+ISO, *^α^*CC100+ISO compared to CCNP100+ISO, *^β^*CC150+ISO compared to CCNP150+ISO, and *^μ^*CC200+ISO compared to CCNP200+ISO.

Serum levels of MMP-2 and MMP-9 increased after the induction of MI (Table 6). All doses of CC and CCNP prevented the increase in MMP-2 with a significantly better effect of CCNP compared to CC (*p* ≤ 0.0027, Table 6 and Figure 5(a)). The best dose of CC to prevent MMP-2 and MMP-9 elevation is 200 mg/kg bw (Table 6 and Figures 5(a) and 5(b)). A similar result was also found for CCNP (Table 6 and Figures 5(a) and 5(b)). The CCNP performed better than CC in preventing the increase in MMP-9 in doses of 100 mg/kg bw and 200 mg/kg bw (*p* < 0.0127, Table 6 and Figure 5(b)).

### 3.5. Light Microscopic Changes of the Myocardium

**Figure 6:**
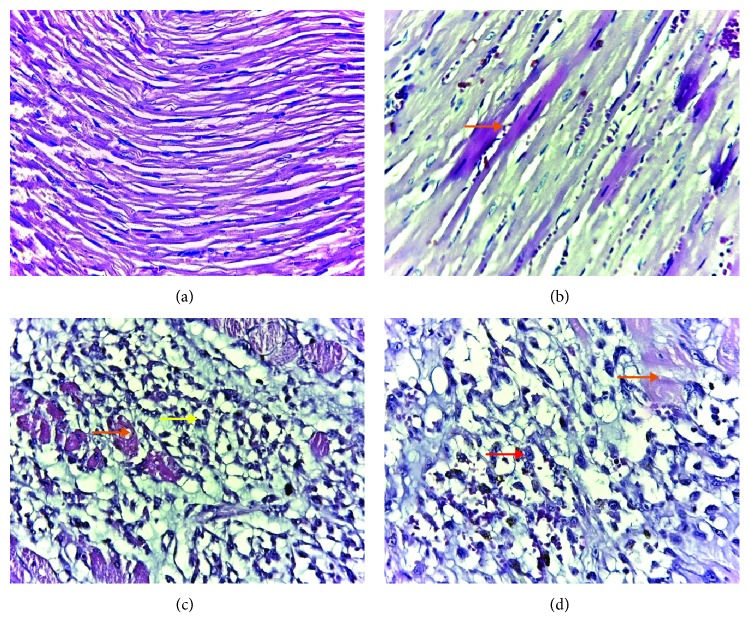
Histopathology on the basis of severity of changes: (a) grade 1—normal myocardial tissue, (b) grade 2—focal myocardial fiber necrosis (orange arrow), (c) grade 3—focal myocardial fiber necrosis (orange arrow) with associated inflammation (yellow arrow), and (d) grade 4—extensive or multifocal myocardial fiber necrosis (orange arrow) with extensive associated inflammation (red arrow).

The histopathological examinations were scored on the basis of severity of changes: grade 1 (intact and homogenous histoarchitecture of the myocardium, Figure 6(a)), grade 2 (focal myocardial fiber necrosis as hypereosinophilic fibers, Figure 6(b)), grade 3 (focal myocardial fiber necrosis with associated interstitial edema and neutrophil infiltration, Figure 6(c)), and grade 4 (extensive or multifocal myocardial fiber necrosis with interstitial edema and hemorrhage with marked neutrophil granulocytes, characterizing acute extensive myofibrillary degeneration, Figure 6(d)). In the study groups, histological changes were observed as follows: in the control group (C), all rates had grade 1; in ISO without any pretreatment (ISOC) group, 6 rats had grade 4 and just one rat had grade 3; in groups treated with curcumin solution, in doses of 100 mg/kg bw (CC100), 150 mg/kg bw (CC150), and 200 mg/kg bw (CC200), and curcumin nanoparticle solution in the dose of 100 mg/kg bw (CCNP100), 3 rats had grade 4 and 4 rats had grade 3; in the group treated with curcumin nanoparticle solution in the dose of 150 mg/kg bw (CCNP150), all rats had grade 3; and in the group pretreated with curcumin nanoparticle solution in the dose of 200 mg/kg bw (CCNP200), 6 rats had grade 3 and 1 rat had grade 2.

## 4. Discussions

In the present study, the ISO-induced MI was confirmed by the elevated serum levels of CK and CK-MB enzymes. CK is an enzyme that is found not only in the cardiac muscle but also in the skeletal muscle. It has an increased serum activity following MI within 6 hours and a peak level on an average at 24 hours and returns to normal values within 2-3 days [19]. CK has three isoenzymes: MM (CK-MM, the skeletal muscle fraction), MB (CK-MB, the cardiac muscle fraction), and BB (CK-BB, the brain fraction). Previously, the total CK was assessed for myocardial infarction, but since the total CK contains 95% of the CK-MM fraction, it is not used as a specific tool in MI [20]. The CK-MB rises in the serum following the same pattern as CK. One advantage of CK-MB over the troponins is the early clearance that helps in the detection of reinfarction [20]. Our results show that pretreatment with all doses of CC and CCNP significantly reduced CK-MB leakage from cardiomyocytes, with the best result for CCNP. These results confirm the cardioprotective effects of curcumin on cardiac myocytes since curcumin was shown to have a membrane-stabilizing action by inhibiting the release of beta-glucuronidase from nuclei, mitochondria, lysosome, and microsome [21]. CCNP had better effects because the nanoparticles provide a more precise delivery of small molecule of curcumin compound in the endocardial layer of the heart and thus exert a significant cardioprotective effect in the myocardium [22].

Our results demonstrate that pretreatment with curcumin and curcumin nanoparticles has antioxidative effects in ISO-induced MI; CC and CCNP prevented the elevation in MDA, TOS, and NOx. CCNP performed better in preventing the elevation of the studied prooxidant parameters (Table 3 and Figures 2(a)–2(c)). The inorganic nitrites and nitrates (NOx), stable end metabolites of NO, were measured in order to evaluate the NO production, a biomarker of nitrooxidative stress [23]. The high concentration of NOx found in the groups with ISO-induced MI compared to the control group (Table 3 and Figure 2(a)) demonstrates the increase of NO synthesis as a response to myocardial infarction, with the activation of the high-output inducible NOS/NO pathway [5]. High levels of iNOS-derived NO contribute to the formation of peroxynitrite, which subsequently leads to significantly increased oxidative stress [24] and severe myocardial apoptosis [25], further leading to an extension of myocardial infarct size [26]. Curcumin has been reported to inhibit nitric oxide synthase activity [27]. The administration of curcumin encapsulated in nanocarriers increases the antioxidant effect compared to that of conventional curcumin as previously demonstrated [28, 29].

The improvement of TOS (Figure 2(c) and Table 3) and TAC (Figure 3(b) and Table 4) parameters was recorded in all our study groups, with more significant results obtained for curcumin nanoparticles (CCNP). The improvement of TAC and the reduction of TOS in the curcumin-treated and curcumin nanoparticle-treated groups was also previously demonstrated in an experimental migraine model in rats [30]. TAC was found to be low in patients with myocardial infarction, and thus the antioxidant therapy may be beneficial in coronary artery disease prevention [31]. TOS was reported to increase in patients with chronic ischemic heart failure [32]. Thiols play a significant role, along with other antioxidants in the body, in mitigating the lipid peroxidative effects of reactive oxygen species (ROS) [33]. A decrease in total thiols in patients with myocardial infarction indicates an increased consumption of thiols due to the increased generation of ROS secondary to ischemia and reperfusion [34]. Thiol levels were significantly increased in the study groups, especially for the groups that received curcumin nanoparticles (Figure 3(a)). Curcumin can increase thiol levels by inhibiting the NF-kappa B activation and induction of glutathione biosynthesis [35]. MDA, a stable metabolite of ROS, is another marker of oxidative stress produced as a byproduct of polyunsaturated fatty acid peroxidation and arachidonic acid metabolism [36]. MDA may accumulate in MI due to the low oxygen level and oxidative stress induced by acute ischemic injury [37]. Its plasmatic level rises immediately after myocardial infarction due to oxidative stress induced by acute ischemic injury [34]. Curcumin prevents MDA elevation by reducing the H_2_O_2_-induced lipid peroxidation [38]. The antioxidative effect of the CC increases with the increase in the dose, as demonstrated in our study, while any of the CCNP doses used in the study provided the highest antioxidant protection compared to conventional curcumin. Our results demonstrate that curcumin nanoparticles exert better antioxidative effects on MI compared to conventional curcumin, thus improving myocardial function more effectively and limiting the extension of heart damage. This result can be explained by the increased metabolic stability of curcumin nanoparticles, better tissue distribution, and enhanced antioxidative properties [39]. The pretreatment with curcumin-nisin-based polylactic acid nanoparticle proved to prevent ISO-induced myocardial infarction in guinea pigs due to the ability of curcumin nanoparticles to increase the activity of the cardiac antioxidant defense [40].

The pretreatment with curcumin and curcumin nanoparticles ensures a significantly lower level of inflammatory cytokines such as TNF-*α*, IL-6, IL-1*α*, IL-1*β*, and RANTES after ISO-induced MI as demonstrated by our study. CCNP performed better compared to conventional curcumin in preventing the increase in the levels of cytokines mentioned above (Table 5 and Figures 4(a), 4(d), and 4(f)). Only the highest CCNP doses used in our study prevented MCP-1 elevation after MI (Table 5 and Figure 4(e)). TNF-*α* and IL-6 are proinflammatory cytokines involved in the synthesis of collagen and scar formation after acute myocardial infarction [41, 42]. TNF-*α* is not expressed in normal cardiomyocytes, but after myocardial infarction, the ischemia and anoxia activate cardiomyocytes and myocardial mononuclear macrophages, which will produce large amounts of TNF-*α* in the myocardium in the infarcted zone and the infarction border zone [43]. Serum levels of IL-6 increase after acute myocardial infarction, and since high IL-6 and C-reactive protein levels coincide with peak cardiac troponin, they could confirm the connection between inflammation and infarct size [44]. In myocardial ischemia, serum levels of IL-1*β* are increased, and they cause the activation of the myofibroblasts involved in cardiac remodeling and the alteration of systolic function after acute myocardial infarction [45–47]. The reduction of the IL-1*β* serum level is associated with a smaller area of the affected myocardial tissue, which explains the role of IL-1*β* in the pathophysiology of acute myocardial infarction [45, 48].

The expression of IL-6, TNF-*α*, and IL-1*β* cytokines is also stimulated by interleukin-1*α* [49] whose release from myocardial cells is stimulated by hypoxia and the acidosis accompanying ischemia [43, 50]. IL-1*α*, released from necrotic cardiomyocytes, may serve as a signal, implicated in the activation of the postinfarction inflammatory response that contributes to adverse cardiac remodeling [51]. It has been suggested that the release of constitutive IL-1*α* may extend ischemic myocardial injury by increasing apoptosis of cardiomyocytes [52]. In patients with myocardial infarction, a significant increase in the serum level of RANTES was previously reported [53, 54], and the elevated serum levels of this marker are associated with a 2 to 3.4 times higher mortality risk in patients with acute coronary syndrome [55].

Administration of curcumin was proved to be effective in limiting the serum level of TNF-*α*, IL-6, IL-1*α*, and IL-1*β* in myocardial ischemia-reperfusion injury in rats [56]. One explanation for this is that curcumin can reduce the ongoing reperfusion injury mediated through inflammatory responses by interfering with NF-*κ*B activation; this pathway is critical in the regulation of transcription of proinflammatory-related genes [27]. Curcumin pretreatment was also proved to be useful in attenuating the expression of MCP-1 in cardiomyocytes after cardiac ischemia-reperfusion injury [57]. The effect of curcumin in reducing RANTES production was reported in spinal cord experimental studies [58, 59]. To our knowledge, no other study focused on the effect of curcumin on the RANTES plasma level in myocardial infarction was published so far. The marked reduction in the serum level of TNF-*α*, IL-6, IL-1*α*, IL-1*β*, and RANTES after ISO-induced MI in subjects with CCNP pretreatment indicates the enhanced anti-inflammatory effect of the curcumin nanoparticles. The effect observed on CCNP can be explained by a higher bioavailability of the nanoformulation, attributed to the direct uptake of nanoparticles through the gastrointestinal tract and their decreased degradation and clearance [60].

CCNP performs better compared to CC in preventing the increase in MMP-2 and MMP-9 levels after ISO-induced MI in rats as demonstrated by our study. MMPs are essential proteolytic enzymes involved in extracellular matrix degradation and structural changes of cardiomyocytes in both the infarcted and noninfarcted myocardium, a process known as cardiac remodeling, which constitutes the anatomic substrate for developing congestive heart failure and sudden cardiac death [61]. MMP-2 and MMP-9 were studied for their roles in left ventricular remodeling and postmyocardial infarction prognosis since they are activated within the myocardial tissue after MI [62, 63]. MMP-2, or gelatinase A, is found in nearly all cell types and degrades collagen type IV, a significant component of the basement membrane, and denatured collagen, as well as other extracellular matrix proteins [64]. MMP-2 impairs the cardioprotective response to oxidative stress via disturbed mitochondrial respiration and excessive lipid peroxidation as demonstrated in myocardial infarction in mice [65]. In acute myocardial ischemia, MMP-9 within the infarcted tissue is derived from neutrophils and may act directly on the ventricular tissue as a protease, but it may also facilitate neutrophil infiltration and degranulation and exacerbate the ischemic insult [66]. The inhibition of MMP-2 is associated with less left ventricular adverse remodeling and higher survival after acute myocardial infarction in mice [63]. MMP-9 inhibition leads to a lower incidence of myocardial rupture after acute myocardial infarction and lowers left ventricular dilation due to less collagen reorganization in the infarcted area in mice [67]. Curcumin treatment inhibits both MMP-2 and MMP-9 through its potent antioxidant action, promoting cardiac repair and ameliorating cardiac dysfunction following myocardial infarction [9]. Curcumin pretreatment was proved to reduce MMP-2 and MMP-9 expression in extracellular matrix degradation after myocardial infarction, by inhibiting the expression of angiotensin II [11]. Curcumin nanoparticles proved effective in reducing the level of MMP-2 in rats with diabetes mellitus, so they can be used as adjuvant treatment for reducing the vascular complication of diabetes mellitus [68]. Our study is the first to report the effect of curcumin nanoparticle pretreatment on MMP-2 and MMP-9 expression in myocardial infarction. Several experimental studies have shown that curcumin pretreatment improves systolic dysfunction and prevents cardiac remodeling [9, 11, 12]. The cardioprotective effect of curcumin results from the attenuation of oxidative stress and the reduced activity of active matrix metalloproteinases [9, 11]. Other effects of curcumin are the inhibition of differentiation of cardiac fibroblasts and the maintenance of the balance between collagen degradation and synthesis [9, 11]. Curcumin nanoparticles exert better effects of curcumin probably due to their increased solubility, resistance to degradation by enzymes, and reduced toxicity [40].

No significant differences were found between the groups pretreated with curcumin or the lowest dose of curcumin nanoparticles regarding the histopathological changes, but better results were obtained for the highest dose of curcumin nanoparticles. The ability of curcumin to reduce the intensity of apoptosis and therefore decrease cardiomyocytes injury after MI by controlling the intensity of proinflammatory response with downregulation of three genes (peroxisome proliferator-activated receptor-*γ*, Bcl-2, and NF-*κ*B) had been reported [15, 69]. Even more, Garvin and coauthors revealed an inherent potency of curcumin to reduce the myocardial infarcted area by modulating immune cell filtration rate and improving the mitochondrial function of the injured cardiomyocytes [70]. The results of our study are consistent with those of Rahnavard and coauthors [69], with the reduction of cardiomyocyte necrosis, edema formation, and infiltration of inflammatory cells compared to the ISO-induced MI group after curcumin administration. Curcumin nanoparticles seem to be more biologically effective than conventional curcumin due to improved absorption, transportation, and bioavailability offering a better delivery of a cardioprotective drug to the infarcted heart [40]. Our results show that curcumin nanoparticle pretreatment can prevent damaged myocardial tissue after MI induction; thus, they could be a viable solution to the preventive strategies in cardiovascular diseases.

## 5. Conclusions

The results of our study demonstrate that curcumin nanoparticles possess cardioprotective effects due to their ability to enhance antioxidant response and to reduce serum levels of proinflammatory cytokines and MMP expression in ISO-induced myocardial ischemia. Curcumin nanoparticles exert better antioxidative effects on MI compared to conventional curcumin, after oral administration, which can lead to improved myocardial function and attenuated heart damage after myocardial ischemia. These results provide new insights into the development of targeted preventive therapies for cardiovascular diseases.

## Figures and Tables

**Table 1:** Design of the experimental myocardial infarction: curcumin and curcumin nanoparticles.

Group no.	Group abb. (description)	ISO (mg/kg bw s.c.)	Pretreatment (mg/kg bw)
1	C (control group)	None	None
2	ISOC (MI control group)	100	None
3	CC100+ISO (100 mg curcumin (CC) with MI)	100	100
4	CC150+ISO (150 mg CC with MI)	100	150
5	CC200+ISO (200 mg CC with MI)	100	200
6	CCNP100+ISO (100 mg curcumin nanoparticles (CCNP) with MI)	100	100
7	CCNP150+ISO (150 mg CCNP with MI)	100	150
8	CCNP200+ISO (200 mg CCNP with MI)	100	200

**Table 2:** Serum levels of myocardial infarction enzymes (values expressed as mean (standard deviation)).

Group abb.	CK (U/l)	CK-MB (U/l)
C	59.00 (10.05)	8.14 (1.07)
ISOC	160.00 (13.54)	28.86 (3.13)
CC100+ISO	126.14 (4.81)	19.14 (1.35)
CC150+ISO	119.00 (1.91)	17.14 (1.35)
CC200+ISO	115.00 (3.27)	16.43 (1.90)
CCNP100+ISO	106.00 (2.58)	14.00 (1.62)
CCNP150+ISO	84.86 (10.21)	13.14 (1.95)
CCNP200+ISO	64.86 (6.47)	11.29 (1.38)

CK = creatine kinase; CK-MB = creatine kinase-MB; C = control; ISOC = isoproterenol without any pretreatment; CC = curcumin solution, in doses of 100 mg/kg bw (CC100), 150 mg/kg bw (CC150), and 200 mg/kg bw (CC200); CCNP = curcumin nanoparticle solution, in doses of 100 mg/kg bw (CCNP100), 150 mg/kg bw (CCNP150), and 200 mg/kg bw (CCNP200).

**Table 3:** Quantification of oxidative stress intensity per group (values expressed as mean (standard deviation)).

Group abb.	NOx (*μ*mol/l)	MDA (nmol/l)	TOS (*μ*mol H_2_O_2_ equiv./l)
C	25.86 (2.34)	1.78 (0.13)	17.43 (1.72)
ISOC	41.00 (3.46)	3.09 (0.18)	47.57 (5.19)
CC100+ISO	36.71 (2.69)	2.78 (0.04)	35.71 (2.29)
CC150+ISO	33.43 (1.51)	2.57 (0.03)	26.71 (1.50)
CC200+ISO	32.71 (2.21)	2.34 (0.08)	21.85 (3.13)
CCNP100+ISO	31.14 (3.72)	2.09 (0.05)	21.00 (2.00)
CCNP150+ISO	30.29 (5.35)	1.84 (0.08)	19.14 (1.35)
CCNP200+ISO	28.43 (1.72)	1.78 (0.05)	18.57 (1.62)

NOx = the indirect assessment of NO synthesis; MDA = malondialdehyde; TOS = total oxidative status; C = control; ISOC = isoproterenol without any pretreatment; CC = curcumin solution, in doses of 100 mg/kg bw (CC100), 150 mg/kg bw (CC150), and 200 mg/kg bw (CC200); CCNP = curcumin nanoparticle solution, in doses of 100 mg/kg bw (CCNP100), 150 mg/kg bw (CCNP150), and 200 mg/kg bw (CCNP200).

**Table 4:** Quantification of the antioxidant capacity per group (values expressed as mean (standard deviation)).

Group abb.	Thiol (mmol/l)	TAC (mmol Trolox/l)
C	0.56 (0.05)	1.16 (0.03)
ISOC	0.31 (0.05)	0.87 (0.09)
CC100+ISO	0.37 (0.02)	0.95 (0.02)
CC150+ISO	0.39 (0.01)	1.03 (0.01)
CC200+ISO	0.42 (0.04)	1.07 (0.01)
CCNP100+ISO	0.43 (0.01)	1.09 (0.02)
CCNP150+ISO	0.44 (0.03)	1.11 (0.02)
CCNP200+ISO	0.47 (0.02)	1.14 (0.02)

TAC = total antioxidant capacity; C = control; ISOC = isoproterenol without any pretreatment; CC = curcumin solution, in doses of 100 mg/kg bw (CC100), 150 mg/kg bw (CC150), and 200 mg/kg bw (CC200); CCNP = curcumin nanoparticle solution, in doses of 100 mg/kg bw (CCNP100), 150 mg/kg bw (CCNP150), and 200 mg/kg bw (CCNP200).

**Table 5:** Serum levels of cytokines per group (values expressed as mean (standard deviation)).

Group abb.	TNF-*α* (ng/ml)	IL-6 (ng/ml)	IL-1*α* (ng/ml)	IL-1*β* (ng/ml)	MCP1 (ng/ml)	RANTES (ng/ml)
C	0.44 (0.05)	0.31 (0.02)	0.40 (0.06)	0.51 (0.04)	0.25 (0.05)	1.85 (0.08)
ISOC	2.47 (0.08)	0.38 (0.03)	0.55 (0.05)	1.13 (0.07)	0.38 (0.05)	3.00 (0.04)
CC100+ISO	1.64 (0.11)	0.37 (0.02)	0.49 (0.02)	1.04 (0.06)	0.35 (0.03)	2.68 (0.06)
CC150+ISO	1.42 (0.12)	0.35 (0.02)	0.47 (0.02)	0.84 (0.05)	0.35 (0.05)	2.56 (0.07)
CC200+ISO	1.14 (0.07)	0.34 (0.01)	0.46 (0.03)	0.78 (0.05)	0.33 (0.04)	2.39 (0.09)
CCNP100+ISO	0.76 (0.10)	0.34 (0.02)	0.44 (0.03)	0.71 (0.08)	0.32 (0.03)	2.29 (0.09)
CCNP150+ISO	0.60 (0.08)	0.33 (0.02)	0.43 (0.02)	0.65 (0.04)	0.31 (0.05)	2.15 (0.09)
CCNP200+ISO	0.51 (0.08)	0.32 (0.02)	0.41 (0.02)	0.54 (0.03)	0.30 (0.05)	2.00 (0.07)

TNF-*α* = tumor necrosis factor alpha; IL-6 = interleukin 6; IL-1*α* **=** interleukin 1 *α*; IL-1*β* = interleukin 1*β*; MCP1 = monocyte chemoattractant protein-1; RANTES = regulated upon activation, normal T cell expressed, and secreted; C = control; ISOC = isoproterenol without any pretreatment; CC = curcumin solution, in doses of 100 mg/kg bw (CC100), 150 mg/kg bw (CC150), and 200 mg/kg bw (CC200); CCNP = curcumin nanoparticle solution, in doses of 100 mg/kg bw (CCNP100), 150 mg/kg bw (CCNP150), and 200 mg/kg bw (CCNP200).

**Table 6:** Serum levels of matrix metalloproteinases per group (values expressed as mean (standard deviation)).

Group abb.	MMP-2 (ng/ml)	MMP-9 (ng/ml)
C	86.00 (8.47)	15.57 (1.27)
ISOC	196.86 (13.13)	24.43 (2.15)
CC100+ISO	142.00 (9.59)	22.71 (1.38)
CC150+ISO	132.00 (4.55)	21.29 (2.21)
CC200+ISO	129.14 (4.98)	20.57 (1.51)
CCNP100+ISO	113.43 (11.84)	20.14 (1.07)
CCNP150+ISO	110.00 (8.10)	19.86 (2.54)
CCNP200+ISO	98.14 (6.74)	18.29 (1.11)

MMP-2 = matrix metalloproteinase-2; MMP-9 = matrix metalloproteinase-9; C = control; ISOC = isoproterenol without any pretreatment; CC = curcumin solution, in doses of 100 mg/kg bw (CC100), 150 mg/kg bw (CC150), and 200 mg/kg bw (CC200); CCNP = curcumin nanoparticle solution, in doses of 100 mg/kg bw (CCNP100), 150 mg/kg bw (CCNP150), and 200 mg/kg bw (CCNP200).

## Data Availability

The experimental data will not be publicly available until the associated Ph.D. thesis is published but can be obtained upon request addressed to Paul-Mihai Boarescu (e-mail: boarescu.paul@umfcluj.ro).

## Supplementary Materials

Supplementary Materials Supplementary Table 1: *p* values for comparisons between groups.
