# Supplementary material for: Effects of Curcumin Nanoparticles in Isoproterenol-Induced Myocardial Infarction
Source: Oxid Med Cell Longev. 2019 May 7;2019:7847142. doi: 10.1155/2019/7847142 (PMC6530192; doi:10.1155/2019/7847142)
Supplement: Supplementary Materials — Supplementary Table 1: p values for comparisons between groups. [file 7847142.f1.docx]

# Effects of curcumin nanoparticles in isoproterenol-induced myocardial infarction

Paul-Mihai Boarescu, Ioana Chirilă, Adriana E. Bulboacă, Ioana Corina Bocșan, Raluca Maria Pop, Dan Gheban, and Sorana D. Bolboacă

**Supplementary Table 1**. P-values for comparisons between groups.

|  | ISOC vs. | | | | | | | CCxxx+ISO vs. CCyyy+ISO | | | CCNPxxx+ISO vs. CCNPyyy+ISO | | | CCxxx+ISO vs.  CCNPxxx+ISO | | |
| --- | --- | --- | --- | --- | --- | --- | --- | --- | --- | --- | --- | --- | --- | --- | --- | --- |
|  | C | CC100+  ISO | CC150+  ISO | CC200+  ISO | CCNP100  +ISO | CCNP150  +ISO | CCNP200  +ISO | 100 vs. 150 | 150 vs. 200 | 100 vs. 200 | 100 vs. 150 | 150 vs. 200 | 100 vs. 200 | 100 vs. 100 | 150 vs. 150 | 200 vs. 200 |
|  | a | b | c | d | e | f | g | A | B | C | X | Y | Z | *α* | *β* | *µ* |
|  | | | | | | | | | | | | | | | | |
| CK [U/L] | 0.0017 | 0.0017 | 0.0017 | 0.0017 | 0.0017 | 0.0017 | 0.0017 | 0.0088 | 0.0215 | 0.0022 | 0.0049 | 0.0017 | 0.0017 | 0.0017 | 0.0017 | 0.0017 |
| CK-MB [U/L] | 0.0017 | 0.0017 | 0.0017 | 0.0017 | 0.0017 | 0.0017 | 0.0017 | 0.0253 | 0.0253 | 0.0033 | 0.1102 | 0.0127 | 0.0107 | 0.0127 | 0.0040 | 0.0033 |
|  | | | | | | | | | | | | | | | | |
| NOx [μmol/L] | 0.0017 | 0.0253 | 0.0017 | 0.0027 | 0.0017 | 0.0027 | 0.0017 | 0.0215 | 0.3067 | 0.0253 | 1 | 0.8480 | 0.0298 | 0.0088 | 0.3379 | 0.0040 |
| MDA [nmol/L] | 0.0017 | 0.0017 | 0.0017 | 0.0017 | 0.0017 | 0.0017 | 0.0017 | 0.0017 | 0.0017 | 0.0017 | 0.0017 | 0.0736 | 0.0017 | 0.0017 | 0.0017 | 0.0017 |
| TOS [μmol H_2_O_2_ equiv./L] | 0.0017 | 0.0017 | 0.0017 | 0.0017 | 0.0017 | 0.0017 | 0.0017 | 0.0017 | 0.0152 | 0.0017 | 0.0476 | 0.4822 | 0.0298 | 0.0017 | 0.0017 | 0.0298 |
| Thiol [mmol/L] | 0.0017 | 0.0152 | 0.0060 | 0.0040 | 0.0017 | 0.0017 | 0.0017 | 0.0476 | 0.1797 | 0.0152 | 0.1797 | 0.0298 | 0.0022 | 0.0017 | 0.0027 | 0.0152 |
| TAC [mmol Trolox/L] | 0.0017 | 0.1417 | 0.0022 | 0.0017 | 0.0017 | 0.0017 | 0.0017 | 0.0017 | 0.0017 | 0.0017 | 0.1797 | 0.0088 | 0.0027 | 0.0017 | 0.0017 | 0.0017 |
|  | | | | | | | | | | | | | | | | |
| TNF-α [ng/ml] | 0.0017 | 0.0017 | 0.0017 | 0.0017 | 0.0017 | 0.0017 | 0.0017 | 0.0088 | 0.0017 | 0.0017 | 0.0073 | 0.0350 | 0.0017 | 0.0017 | 0.0017 | 0.0017 |
| IL-6 [ng/ml] | 0.0017 | 0.7983 | 0.0350 | 0.0060 | 0.0127 | 0.0033 | 0.0017 | 0.1417 | 0.2502 | 0.0350 | 0.3711 | 0.3711 | 0.0967 | 0.0409 | 0.0409 | 0.0253 |
| IL-1α [ng/ml] | 0.0027 | 0.0152 | 0.0040 | 0.0022 | 0.0017 | 0.0017 | 0.0017 | 0.3379 | 0.2248 | 0.0409 | 0.3379 | 0.1417 | 0.0409 | 0.0073 | 0.0127 | 0.0088 |
| IL-1β [ng/ml] | 0.0017 | 0.0350 | 0.0017 | 0.0017 | 0.0017 | 0.0017 | 0.0017 | 0.0017 | 0.0553 | 0.0017 | 0.0736 | 0.0017 | 0.0049 | 0.0017 | 0.0017 | 0.0017 |
| MCP1 [ng/ml] | 0.0027 | 0.1599 | 0.1599 | 0.0553 | 0.0298 | 0.0409 | 0.0127 | 0.5653 | 0.7015 | 0.2502 | 0.4062 | 0.6093 | 0.5229 | 0.0736 | 0.2774 | 0.2248 |
| RANTES [ng/ml] | 0.0017 | 0.0017 | 0.0017 | 0.0017 | 0.0017 | 0.0017 | 0.0017 | 0.0073 | 0.0088 | 0.0017 | 0.0350 | 0.0088 | 0.0017 | 0.0017 | 0.0017 | 0.0017 |
|  | | | | | | | | | | | | | | | | |
| MMP-2[ng/ml] | 0.0017 | 0.0017 | 0.0017 | 0.0017 | 0.0017 | 0.0017 | 0.0017 | 0.0409 | 0.3379 | 0.0127 | 0.6093 | 0.0181 | 0.0253 | 0.0027 | 0.0017 | 0.0017 |
| MMP-9[ng/ml] | 0.0017 | 0.1252 | 0.0298 | 0.0060 | 0.0033 | 0.0073 | 0.0017 | 0.1797 | 0.6093 | 0.0298 | 0.7494 | 0.2248 | 0.0152 | 0.0060 | 0.3067 | 0.0127 |
